# Keeping Pace with the History of Evolving Runtime Models

**DOI:** 10.1007/978-3-030-71500-7_13

**Published:** 2021-02-24

**Authors:** Lucas Sakizloglou, Matthias Barkowsky, Holger Giese

**Affiliations:** 8 Universidad Autónoma de Madrid, Madrid, Spain; 9 University of Twente, Enschede, The Netherlands; Hasso Plattner Institute, University of Potsdam, Potsdam, Germany

**Keywords:** runtime models, time-awareness, temporal graph queries

## Abstract

Structural runtime models provide a snapshot of the constituents of a system and their state. Capturing the history of runtime models, i.e., previous snapshots, has been shown to be useful for a number of aims. Handling, however, history at runtime poses important challenges to tool support. We present the InTempo tool which is based on the Eclipse Modeling Framework and encodes runtime models as graphs. Key features of InTempo, such as, the integration of temporal requirements into graph queries, the in-memory storage of the model, and a systematic method to contain the model’s memory consumption, intend to address issues which seemingly place limitations on the available tool support. InTempo offers two operation modes which support both runtime and postmortem application scenarios.

## 1 Introduction to InTempo

A (structural) *Runtime Model* (RTM) provides a snapshot of the constituents of a system and their state [3]. RTMs are typically employed in the context of *Self-adaptive Systems* (SAS) [4], where a feedback loop adapts the system behavior at runtime in response to external or internal stimuli, the latter represented as model fragments in the RTM and detected via the execution of model queries.

Encoding an RTM as a graph enables detection via *graph queries*, which specify a sought (graph) pattern. Such an encoding conforms to a *metamodel* which restricts the structure of model instances and defines types of vertices, edges, and attributes. Formally, these concepts rely on *typed, attributed graph transformation* [6] where graphs are typed over a *type graph*.

Capturing the *history* of RTMs, i.e., previous snapshots, may be useful for a number of aims such as the detection of recurrent behavior or postmortem analysis [3,8]. However, handling history at runtime poses important challenges to tool support. Tools are required to enable the specification and timely execution of queries with *temporal requirements*, i.e., requirements on the evolution of patterns over multiple snapshots. Timely execution is crucial for SAS, where a loop may depend on query results before planning and performing adaptations.

Faced with these challenges, the available tool support is seemingly limited either by the lack of support for direct specification of temporal requirements in graph queries [5] or by the on-disk representation of the model [8,11] that introduces an overhead on execution times in runtime settings, e.g., in SAS.

We present the InTempo (Incremental queries with TEMPOral requirements) tool (available online at [13]) which is based on the eponymous querying scheme in [15] and aims at mitigating these limitations. InTempo introduces ITQL, a language for the specification of *temporal graph queries*, which allow for the expression of temporal requirements. The core functionality of InTempo executes a query over an in-memory RTM which captures information about previous snapshots, called *Runtime Model with History* ($\mathrm{RTM^H}$), and returns the pattern occurrences in the $\mathrm{RTM^H}$ that satisfy the specified temporal requirements. InTempo is implemented in the Eclipse Modeling Framework (EMF) [7] and can be used either via the Eclipse user interface or via an API. The latter enables InTempo results to be utilized by other tools, e.g., a SAS feedback loop.

InTempo offers two operation modes intended for different application scenarios (see Figure 1 for an illustration). The *$RTM^H$Analysis* (Section 2) constitutes the core functionality of InTempo and executes a user-specified ITQL query (in a file with *.itql* extension—required extensions are in parentheses in Figure 1) over a user-provided $\mathrm{RTM^H}$, i.e., a persisted instance of an EMF model (in the standard *xmi* format). This mode returns the query results for the given $\mathrm{RTM^H}$. Query results are kept in-between analyses and are updated by each $\mathrm{RTM^H}$Analysis, which is also known as *incremental (query) execution*. The $\mathrm{RTM^H}$Analysis is intended to be used in settings where query results can be further utilized at runtime. For instance, a SAS feedback loop may use InTempo to detect problems formulated as patterns, similarly to [9]. Subsequently, the query results may be utilized to plan adaptations which address these problems.

The *LogAnalysis* operation mode (Section 3) assumes that, instead of being captured by an $\mathrm{RTM^H}$, past and present data about the system have been captured in an event log. InTempo introduces E2P, a specification language that allows for the mapping of event types to corresponding modifications of model fragments, i.e, nodes, edges, and attributes. As input, LogAnalysis requires the ITQL query, the log (with comma-separated values), and the E2P mapping. It then processes the log and maintains an internal $\mathrm{RTM^H}$ which it uses to perform $\mathrm{RTM^H}$Analysis upon every event. LogAnalysis is intended for postmortem scenarios. Thus, it returns the results that were valid after each $\mathrm{RTM^H}$Analysis sorted by the log timestamps, which affords a global, yet detailed, view on the evolution of the system state.

InTempo is capable of containing the data accumulation in the $\mathrm{RTM^H}$ by systematically discovering and discarding data that is obsolete with respect to a given timestamp, i.e., not relevant to future query executions—this capability is presented in detail in [15]. Note that an implicit requirement of both operation modes is that the metamodel of the analyzed system has been encoded as an EMF Ecore model and is available in Eclipse (gray input in Figure 1).

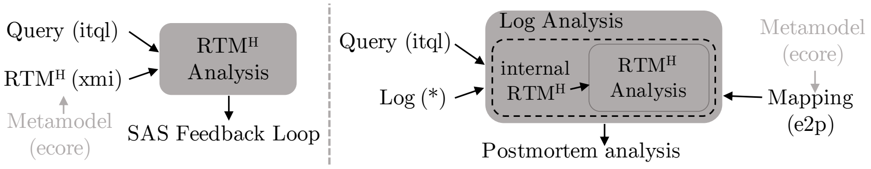

Fig. 1: InTempo Execution Modes and Exemplary Application Scenarios

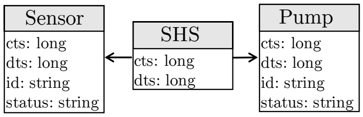

Fig. 2: SHS Metamodel

**Exemplary Application** To demonstrate the features and operation of InTempo we rely on an example drawn from the case-study conducted in [15]. Based on real-world smart medical environments, the case-study envisions a Smart Healthcare System (SHS) where certain medical procedures are automated and performed by devices, such as a smart pump administering medicine or a sensor tracking patient data and diagnoses—as otherwise a clinician would be doing. Data collected from the SHS are aggregated and recorded in medical (event) logs.

InTempo requires a metamodel which has been instrumented such that all nodes have at least two attributes named `cts` and `dts`, which capture the time point of creation, respectively deletion, of the node in the system. As an example, see the metamodel of our SHS in Figure 2. Note that to encode `cts` and `dts` for edges in EMF, the respective edges would have to be modeled as nodes. Technically, an RTM$^{\mathrm{H}}$ is an instance of such a metamodel. See $\mathrm{G}_3$ in Figure 3 for an example based on the SHS metamodel: The RTM$^{\mathrm{H}}$ reflects that a node of type `Sensor` that is attached to the patient with `id=1` has been activated and thus has been added to the `SHS` at timestamp `3`. The sensor `status` reflects that the patient has been diagnosed with sepsis. The value $\infty$ reflects that a `dts` for this node has not been set, i.e., the node is still present in the modeled system.

## 2 RTM$^{\mathrm{H}}$Analysis

This section presents an exemplary query in ITQL which it then uses to demonstrate the RTM$^{\mathrm{H}}$Analysis. It concludes with technical details.

**InTempo Query Language (ITQL)** Formally, a temporal graph query $q$ is characterized by a (graph) pattern $p$ and an *application condition* $ac$, denoted $q = (p, ac)$. A match $m$ corresponds to an occurrence of $p$ in the RTM$^{\mathrm{H}}$. In order for $m$ to be valid, it must satisfy the $ac$. ITQL supports the formulation of $ac$ in the *Metric Temporal Graph Logic* (MTGL) [10] which supports operators such as negation ($\neg$), existential quantification ($\exists$), conjunction ($\wedge$), and the *metric*, i.e, interval-based, temporal operators *until* ($\mathrm{U}_I$, where $I$ is a time interval over $\mathbb{R}_0^+$) and *since* ($\mathrm{S}_I$), as well as abbreviations such as *eventually*, i.e., $\Diamond_I \exists n = \mathit{true}\, \mathrm{U}_I \exists n$, where $n$ is a graph pattern and *true* is always satisfied. MTGL also supports the *nesting* of patterns to *bind* graph elements in outer conditions and relate them to inner (nested) conditions, i.e., elements common to two patterns $n_1$ and $n_2$ refer to the same element in the RTM$^{\mathrm{H}}$.

MTGL is able to express real-time properties such as *"every patient diagnosed with sepsis, must eventually within 5 time units be given the proper drug"* (adjusted from the medical guideline in [14]). In an RTM$^{\mathrm{H}}$ of the SHS, InTempo can find violations of the property above by executing the ITQL query $q_1 = (n_1, \kappa)$, with $\kappa$ the MTGL formula $\neg(\Diamond_{[0,5]} \exists n_2)$ and $n_1$, $n_2$ patterns representing a sepsis diagnosis and drug administration respectively. The query searches for matches of $n_1$ in the RTM$^{\mathrm{H}}$ that satisfy $\kappa$, i.e., for patients that,

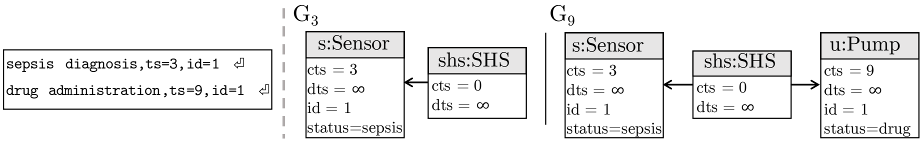

Fig. 3: Exemplary Medical Log and Corresponding RTM$^H$ $G_3$ and $G_9$

although diagnosed with sepsis, did not receive a drug within the designated time. In InTempo, each match is associated with a *temporal validity*, i.e., a set of time intervals for which, based on the overlap among the `cts` and `dts` of the matched elements and the interval for which *ac* is satisfied, the match is valid. ITQL also allows for the definition of OCL constraints [12] on sought patterns.

**Output** The ITQL specification for the query $q_1$ is shown in Figure 4. Performing RTM$^H$Analysis for the query $q_1$ on the RTM$^H$ $G_9$ of Figure 3 returns one match, since there is indeed no `Pump` attached to the `SHS`, i.e., a match for $n_2$, within five time units after a `Sensor` was activated, i.e., a match for $n_1$ was found. The temporal validity interval $[3, 4]$ is returned together with the match. The match, i.e., violation, is indeed valid only for that interval since after timestamp `4`, a match for $n_2$ starts to exist within five time units of a match for $n_1$. If the API of InTempo is used, the query returns the match of the $n_1$ pattern, i.e., the EMF objects, together with the temporal validity. In case InTempo is used via the UI it displays a message box in Eclipse with the following message: `SHS@0[] Sensor@3[status=sepsis] [[3,4]]`. Note that "`@`" precedes the `cts` of an object and values within square brackets are attributes of the object.

```
($n1 , !(true U[0,5] E $n2))

declarations{
  n1{shs:SHS
    s:Sensor
    shs -ownedSensors-> s
    [OCL:"s.status='sepsis'"]}

n2{shs:SHS
    s:Sensor
    u:Pump
    shs -ownedPumps-> u
    shs -ownedSensors-> s
    [OCL:"u.status='drug'"]}
}
```

Fig. 4: Example query in ITQL

**Technical Details** For the execution of temporal graph queries, InTempo employs the operationalization framework presented in [15]. The framework supports the decomposition of a query into a suitable ordering of simpler sub-queries which is executed bottom-up. The outermost query computes the overall result. For pattern-matching, InTempo employs the *Story Diagram Interpreter* from [1] which uses heuristics shown to reduce the pattern-matching effort. InTempo provides an Xtext [2] editor for ITQL which supports completion suggestions for element types and validation of the query syntax.

## 3 LogAnalysis

This section demonstrates the LogAnalysis operation mode which assumes that data from past states have been captured as events in a log. InTempo offers the capability to process the system changes and, upon each change, obtain an updated RTM$^H$ which is then used internally to perform RTM$^H$Analysis.

```
init:{adds shs:SHS}

"sepsis diagnosis":{
    adds s:Sensor [status="sepsis" id=
    *p2]
    adds-ref shs -ownedSensors-> s}

"drug administration":{
    adds u:Pump [status="drug" id=*p2]
    adds-ref shs -ownedPumps-> u}
```

Fig. 5: E2P Example for SHS

**Events-to-Patterns (E2P) Specification Language** The mapping of log events (which encapsulate system changes) to prescribed modifications on an $RTM^H$ is facilitated by E2P. An E2P specification consists of mappings between events and actions that should be performed on an $RTM^H$. E2P supports five actions (formulated as verbs): *adds*, to add a node and optionally assign values to the added node's attributes; *adds-ref*, to add an edge between two nodes; *modifies*, to modify the attribute values of a node; *deletes* and *deletes-ref*, to delete a node, respectively an edge, from the $RTM^H$. To accommodate linked data, E2P allows for the *indexing* of added nodes so that later events can refer to modifications that have been processed earlier. An example of an E2P mapping from an exemplary log in Figure 3 (left) to the corresponding elements of the SHS is shown in Figure 5. Note that edge types, e.g. `OwnedPumps`, are not depicted in Figure 3.

As an example, the event `drug administration` from the medical log in Figure 3 corresponds to the following changes to the (internal) $RTM^H$: a `Pump` is created; its attribute `status` is set to "`drug`" and its attribute `id` takes the value of the second field after the event name (expressed by the special $*p$ token), i.e., the `id` field in the log of Figure 3. By default, the `cts` is set to the value of the event field that is next to the event name, i.e., the `ts` field in Figure 3. The *init* statement is used to initialize the $RTM^H$ and the `cts` of nodes within is set to zero. To increase the readability of specifications, an explicit assignment for the `dts` may be omitted: Unless there is an attribute assignment, the `dts` of all nodes is set to the maximum value supported.

**Output** LogAnalysis provides a view on the matches *per event timestamp*. Performing LogAnalysis on the query $q_1$ and the log of Figure 3 would return:

```
@3 SHS@0[] Sensor@3[status=sepsis] [[3,∞]]
@9 SHS@0[] Sensor@3[status=sepsis] [[3,4]]
```

First, the `sepsis diagnosis` event is processed which makes the internal $RTM^H$ be identical to $G_3$ in the same figure. The query is executed using $RTM^H$Analysis and returns a match, i.e., violation, since at that moment a match for $n_2$ does not exist in the graph. The temporal validity is equal to [3,∞], i.e., the match is valid from time point 3 onward. Next, the `drug administration` event is processed which leads to $G_9$. The result of $RTM^H$ Analysis for $G_9$ is the same as the result described in Section 2.

**Technical Details** In LogAnalysis the query execution framework monitors the $RTM^H$ for changes and, upon every change, recomputes the matches. Previous matches are kept in-between executions and therefore the query is executed incrementally. Similarly to ITQL, E2P is supported by an Xtext editor that offers syntax validation and completion suggestions for element types.

## 4 Conclusion and Future Work

We presented InTempo, an EMF tool which enables the specification and incremental execution of temporal graph queries over a runtime model with history. The latter can be either provided as input or obtained by an event log. InTempo stands out from relevant tools owing to the integration of temporal requirements into graph queries, the in-memory representation of the model, and the systematic measures to contain memory consumption despite the accumulation of temporal data. Moreover, InTempo offers input editors with features that aim at helping the user, e.g. syntax validation. In the future, besides streamlining InTempo, we plan to perform extensive evaluation and comparisons with other tools. Moreover, we plan to explore the utilization of InTempo in self-adaptation scenarios where the history of the system is required.
